# In Memoriam: Steve Wing

**DOI:** 10.1289/EHP1406

**Published:** 2017-01-01

**Authors:** Virginia T. Guidry

**Affiliations:** Virginia T. Guidry is an epidemiologist and writer who worked with Steve Wing for 12 years during her master’s, doctoral, and postdoctoral training at UNC-CH.

We lost a beloved scholar, mentor, and environmental justice warrior when Steve Wing passed away on 9 November 2016. He was at home with his family in Pittsboro, North Carolina.

Steve was an epidemiologist who studied topics that other scientists wouldn’t touch, and he did so in collaboration with the people most affected. He documented the health impacts of landfills, industrial-scale livestock production, and sewage sludge applied to rural fields—issues that often disproportionately affect low-income communities of color.

His dedication to highlighting injustices, with unwavering humility, kindness, and grace, won broad respect across North Carolina and around the world. Through community-based participatory research, or CBPR, Steve helped communities educate themselves and gather information they could use to organize and engage with elected officials regarding their concerns.

“Steve helped communities confidently speak up and speak out about issues and what the impact could possibly be on their health and the environment,” said longtime collaborator Naeema Muhammad, co-director and community organizer for the North Carolina Environmental Justice Network (NCEJN). “He was truly ‘the people’s professor.’”

Steve’s diagnosis with advanced cancer in February 2015 stunned his collaborators at the NCEJN and his colleagues at the University of North Carolina at Chapel Hill (UNC-CH), where he was a faculty member in the Department of Epidemiology. Ever committed to fighting injustices, he remained engaged in his work, as his health allowed, even while undergoing chemotherapy. In his last weeks, he was still contributing to several environmental justice–related lawsuits.

“His concern for the way things are kept him working,” said Muhammad. “He wanted to get as much done as he could, for as long as he could.”

Steve was born in New Orleans, Louisiana, on 3 October 1952. As a child, he was moved by the funeral parades he witnessed at the African-American church near his family’s home. Both the music and the racial inequities he observed made a deep impression on his young mind, contributing to a lifelong love of jazz and an equally strong commitment to social justice.

When Steve was 12, his family moved to Durham, North Carolina. He attended Vassar College in New York, where he met his wife, Betsy. They returned to North Carolina and built a home in rural Chatham County, where they raised two daughters, Ann and Marion. Steve earned a master’s degree in sociology from Duke University and a PhD in epidemiology from UNC-CH. He joined the UNC-CH faculty soon after, initially studying trends in cardiovascular and occupational health.

Steve’s academic career took a turn when he began studying workers’ health at the nuclear weapons plant at Oak Ridge National Laboratory in Tennessee. He unexpectedly found that the workers’ radiation badge readings were associated with later cancer mortality, but only if the workers were followed long enough for this to become evident (Wing et al. 1991). Other scientists and government officials intensely questioned the results, but Steve reported them anyway.

He learned something critical from the experience—that he could have a public health impact by working on focused topics where community organizations could use the findings.

“No one had ever complained when I wrote a paper about social inequality, but if it was a paper about ionizing radiation, it caused a huge uproar, and it was of interest to labor groups, and even to this day is of interest to workers,” said Steve in a 2015 interview (Schoenbach 2015).

In the 1990s Steve learned of community concerns about large-scale hog farms that were proliferating in eastern North Carolina. He began attending meetings organized by the Concerned Citizens of Tillery (CCT) to understand more about the concentrated animal feeding operations, or CAFOs, as they were called, eventually joining with Gary Grant of CCT and the late Nan Freeland to found the NCEJN in 1997.

Steve and NCEJN began conducting CBPR to document the disproportionate health impacts of CAFOs on communities of color and low-income communities in North Carolina (Wing et al. 2000). He was most proud of the Community Health Effects of Industrial Livestock Operations study, in which adults who lived near CAFOs took part in an intensive 2-week protocol to measure acute health outcomes while instruments on a trailer nearby monitored livestock-related air pollution. There was barely any attrition among the 101 participants, nearly all of whom were African American. The results showed that increases in air pollution were associated with increases in irritation and respiratory symptoms (Schinasi et al. 2011), increases in blood pressure (Wing et al. 2013), and decreases in quality of life (Tajik et al. 2008). Perhaps just as importantly, a qualitative analysis found that the study participants benefited from study participation through the education and community organizing that were part of the design (Wing et al. 2008).

CBPR seemed to come naturally for Steve. He saw the approach as a democratic way to distribute university resources to systematically disadvantaged areas and balance the typical power differential between researchers and research participants. His thoughtful personality, deep respect for others, and comfort with sharing leadership roles made this possible.

These same qualities made Steve a beloved mentor. He was known for asking difficult questions and challenging students to think differently, be it about an epidemiologic study design or how privilege may have influenced their perspectives. When NCEJN organized a celebration for Steve in the fall of 2015 (NCEJN 2015), tributes from current and former students came flooding in.

“When I think about the most important lessons I learned from Steve Wing, they are not lessons that you learn in a textbook,” wrote UNC alumna Jennifer Norton, now in New York City. “I learned the importance of maintaining the integrity of the science so the findings can be used to support and advocate for policy changes to improve people’s lives. I also learned to maintain my integrity as a person and keep doing the right thing, even, and especially, when others may try to dissuade you.”

At this same celebration a year ago, Steve spoke to the packed room about why to fight social and environmental injustices. “We don’t do it because we’re going to win tomorrow or the next day. We do it because this is what we love, and we love each other,” he said. “We’re not here very long, so while we’re here, let’s get together and challenge the injustices. Because what else is more fun than that?”
